# *Malus domestica*: A Review on Nutritional Features, Chemical Composition, Traditional and Medicinal Value

**DOI:** 10.3390/plants9111408

**Published:** 2020-10-22

**Authors:** Jiri Patocka, Kanchan Bhardwaj, Blanka Klimova, Eugenie Nepovimova, Qinghua Wu, Marco Landi, Kamil Kuca, Martin Valis, Wenda Wu

**Affiliations:** 1 Biomedical Research Centre, University Hospital, 50003 Hradec Kralove, Czech Republic; toxicology@toxicology.cz; 2 Faculty of Health and Social Studies, Department of Radiology and Toxicology, University of South Bohemia Ceske Budejovice, 37005 Ceske Budejovice, Czech Republic; 3 Department of Botany, Shoolini University of Biotechnology and Management Sciences, Solan 173229, H.P., India; kanchankannu1992@gmail.com; 4 Department of Neurology of the Medical Faculty of Charles University and University Hospital in Hradec Kralove, Sokolska 581, 50005 Hradec Kralove, Czech Republic; blanka.klimova@uhk.cz (B.K.); martin.valis@fnhk.cz (M.V.); 5 Department of Chemistry, Faculty of Science, University of Hradec Kralove, 50003 Hradec Kralove, Czech Republic; eugenie.nepovimova@uhk.cz (E.N.);; 6 College of Life Science, Yangtze University, Jinzhou 434025, China; 7 Department of Agriculture, Food and Environment, University of Pisa, Via del Borghetto Pisa 80, 56124 Pisa, Italy; marco.landi@unipi.it; 8 MOE Joint International Research Laboratory of Animal Health and Food Safety, College of Veterinary Medicine, Nanjing Agricultural University, Nanjing 210095, China

**Keywords:** apple, bioactive compounds, human health, polyphenol, polysaccharide, phytosterol, triterpene

## Abstract

Fruit-derived bioactive substances have been spotlighted as a regulator against various diseases due to their fewer side effects compared to chemical drugs. Among the most frequently consumed fruits, apple is a rich source of nutritional molecules and contains high levels of bioactive compounds. The main structural classes of apple constituents include polyphenols, polysaccharides (pectin), phytosterols, and pentacyclic triterpenes. Also, vitamins and trace elements complete the nutritional features of apple fruit. There is now considerable scientific evidence that these bioactive substances present in apple and peel have the potential to improve human health, for example contributing to preventing cardiovascular disease, diabetes, inflammation, and cancer. This review will focus on the current knowledge of bioactive substances in apple and their medicinal value for human health.

## 1. Introduction

Apple (*Malus domestica* Borkh.; Rosaceae) is one of the most economically and culturally significant, nutrient-rich fruit grown in all temperate zones [1]. The whole fruit is eatable except seeds; apart from that, many other products are produced from them: Ciders and juices, jams, compotes, tea, wine, or dry apples. They are irreplaceable in human nutrition since they increase immunity, have a positive effect on stress resistance, and they contain many bioactive substances that are beneficial for humans. There is no doubt that apples are healthy and have many health benefits, but it was modern medicine, based on evidence, not experience, which had to prove their usefulness for human health [2].

The research on apple bioactive substances is mainly concentrated in the pulp and peel of an apple. Overall, a large number of bioactive substances including polyphenols, polysaccharides, plant sterols, pentacyclic triterpenes, and organic acids have been found in apples, and it has been shown that their presence significantly differs in pulp and skin (Table 1). From the human health point of view, polyphenols, polysaccharides, plant sterols, and triterpenes especially jointly contribute to the majority of positive effects on human health, such as antioxidants, anti-cancer, and anti-inflammatory [11,12].

This review will summarize the apple bioactive substances including polyphenols, hydroxycinnamic acid, glycosylated flavonoids, flavan-3-ols, proanthocyanidins (PAC), polysaccharides (pectin), phytosterols, pentacyclic triterpenes, triterpene acids, dihydrochalcones, and vitamin. 

Also, many types of antioxidant compounds present in apple and apple peels have been reported such as catechin, chlorogenic acid, epicatechin, cyanidin-3-galactoside, procyanidin, coumaric acid, gallic acid, phloridzin, quercetin-3 galactoside and quercetin-3-rhamnoside. However, in apple flesh, there are some compounds found in a lesser amount, i.e., epicatechin, catechin, phloridzin, and procyanidin than in the peels. In apple, concentrations of compounds have been reported as quercetin glycosides, 13.2 mg/100 g fruit; phloretin glycosides, 5.59 mg/100 g fruit; procyanidin B, 9.35 mg/100 g fruit; chlorogenic acid, 9.02 mg/100 g fruit; vitamin C, 12.8 mg/100 g fruit; epicatechin, 8.65 mg/100 g fruit [13]; However, chlorogenic acid is found higher in the flesh than in the peel [14]. Our main goal was to discuss the functions of these bioactive substances and their use in medicine. Finally, we will cast a future perspective of the apple bioactive substances in disease treatment and the development of functional healthcare food. We hope this review will help to understand the function of apple bioactive substancesand provide some new lights on the drug or functional healthcare food development from bioactive substances in apple.

## 2. Polyphenols

**Figure 1:**
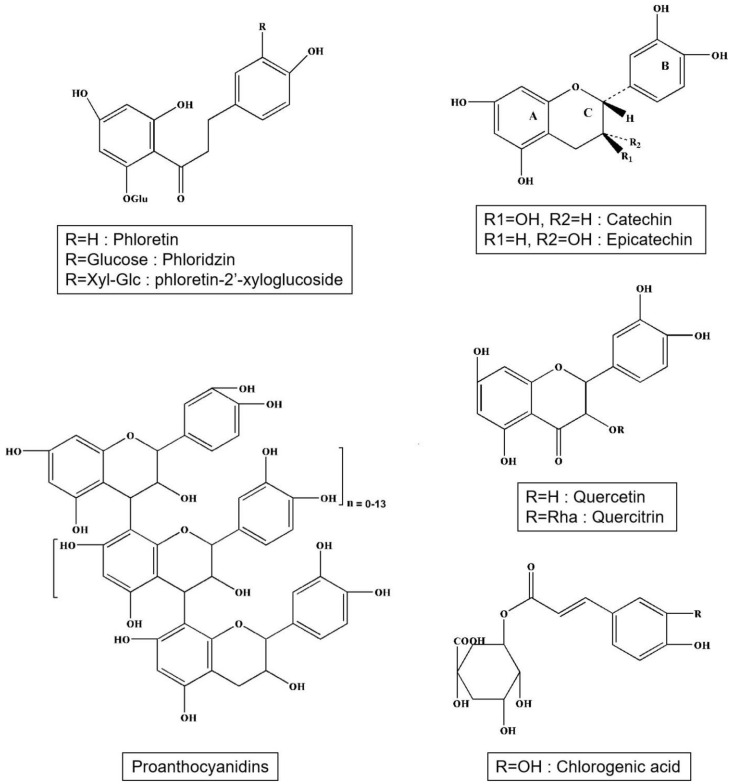
Structures of main phenolic acids, dihydrochalcones and flavonoids in apple.

Apple polyphenols include phenolic acids, dihydrochalcones, and flavonoids (Figure 1). Phenolic acids are mainly chlorogenic acid and caffeic acid (Table 2). Caffeic acid is a derivative of cinnamic acid (Hydroxycinnamic acid). Chlorogenic acid exists in the form of coffee quinic acid in apples, and caffeic acid is produced after hydrolysis. Dihydrochalcones contain phloridzin, phloretin, and phloretin-2′-xyloglucoside. Flavonoids include mainly catechins, epicatechins, proanthocyanidins (B1, B2, B5, C1), quercetin, and quercitrin (Table 3). The average concentrations of phenolic compounds are in Table 4. The profile of polyphenolic substances at different apple varieties and in different parts of the apple are frequently and significantly different [15]. The fruits of the crab apple tree (*Malus prunifolia*) have higher polyphenol content than bred varieties of apples [16]. Epicatechins and procyanidins, gallic acid, protocatechuic, chlorogenic, ferulic and *p*-coumaric, quercetin, and myricetin were detected from this fruit [16]. An American study on eight apple varieties grown in Ontario has revealed that the greatest amount of polyphenols is contained in the cultivars Red Delicious and Northern Spy, and the smallest amount is in the cultivar Empire. The higher concentration of polyphenols was in the peel, but the peel contains, in comparison with apple pulp, a higher concentration of substances from environmental pollution [17]. Altogether, 16 substances from five main polyphenolic groups were discovered. Esters hydroxycinnamic acids, phloretin glycosides, and flavan-3-ols were found both in the pulp and in the peel, while quercetin glycosides were almost exclusively contained in the peel. Cyanidin 3-galactoside was only present in the peel of red apple varieties. Other researchers such as Andre et al. (2012) have analyzed phenolic compounds in 109 apple varieties, and they have discovered that their total amount in the whole fruits ranged between 29 and 7882 mg/kg and in the peel between 733 and 4868 mg/kg of fresh weight. The main phenolic constituents were epicatechins and procyanidins and from triterpenes ursolic acid (44.7 up to 3522 µg/g) and linoleic acid (47.2 up to 838 µg/g) [18]. In many different ancient Tuscan apple varieties such as Nesta, Panaia-red, and Cipolla, phenolic content and antioxidant activity have been studied and found that the Panaia-red have the highest amount of phenolic content and possess good antioxidant activity as compared to the commercial golden delicious variety [19]. Also, the study on ancient apples from Siena revealed that the Solaio’ and ‘Campo Pianacce’ contain highest polyphenol contents (8.72 mg GAE/g FW and 2.25 mg GAE/g FW, good antioxidant potential, and suggest us to take as a functional food [20,21]. The functional foods consumption develops the anti-inflammatory and antioxidant activities in the organism and helps in fighting against cancer and cardiovascular diseases [22].

Apples contain a large number, and high levels, of phenolic compounds with antioxidant activity and given that there is a correlation between the content of phenolic compounds and antioxidant activity among all the varieties of apple [23], it is evident that polyphenols are the chief antioxidant biomolecules of this fruit. In addition to their potent antioxidant effect, apple polyphenols show several other useful pharmacological properties [24]. For instance, apple polyphenols have the potential to be hepatoprotective and inhibit hepatic steatosis [25]. As a feed additive for pigs, apple polyphenols can reduce fat deposits in the liver of finishing pigs, improve lipid distribution, and strengthen the distribution of antioxidant enzymes [26]. Moreover, apple polyphenols are potentially effective dietary supplements and protect the stomach lining from damage by aspirin [27], protect colonic mucosa in ulcerative colitis [28], and reduce inflammatory processes, which lead to bowel disease [29]. Some studies have also shown that apple polyphenols also have the capacity for the elimination of hazardous materials such as lead and mercury in the body [30], antibacterial [31,32] reduction of postprandial glycemia [33], or inhibition of cancer cell proliferation [34,35]

Polyphenols of apples have other effects, including the prevention of degenerative diseases [36]. Due to a favorable effect on the intestinal microflora, apple polyphenols attenuate the risk of inflammatory bowel disease [37,38], metabolic syndrome, and cardiovascular diseases [39,40]. Studies have shown that the total phenolic, flavonoid content of apple peel polyphenol extract (PAP) is significantly higher than that of apple fleshes (PAF). Mice administered with 250 mg/kg of PAP and PAF for 28 days showed lower blood pressure, improved endothelial function, ameliorated lipid homeostasis, and decreased insulin resistance. Furthermore, they inhibit atherosclerosis, and PAP exhibited much more potent cardioprotective effects than PAF in mice [41].

Apple polyphenols are often used as food additives due to their potent antioxidant capacities and antibacterial properties. Apple pomace is rich in polyphenols as a by-product of apple processing. Studies have shown that golden crown apple pomace extract has the highest total flavonoid content of ethyl acetate extract (EAE) [42]. It also showed good antibacterial activity against *Staphylococcus aureus*, the minimum inhibitory concentration (MIC) was 1.25 mg/ml, and the MIC against *E. coli* was 2.5 mg/ml. Phloridzin and phloretin showed better antibacterial activities than the EAE, which were MICs of 0.5 and 0.1 mg/ml against *Staphylococcus aureus*, or MICs of 1.5 and 0.75 mg/ml against *E. coli*, respectively. Apple polyphenols also have a strong inhibitory effect on caries bacteria transglucosylase (GTase), which can prevent the formation of tartar.

Moreover, the main anti-GTase component of apple polyphenols is apple condensed tannin, which has a higher GTase inhibitory capacity than catechin (EGCG) in green tea. It can also be used as a toothpaste additive and plays a vital role in preventing caries and cleaning [10]. Apple polyphenols can also inhibit and eliminate the irritating odors of methyl mercaptan and trimethylamine. When washing fish fillets, adding apple polyphenols at a dose of 250 μg/mL can reduce volatile trimethylamine by 70%. Meanwhile, apple polyphenols also suppress the production of methyl mercaptan, and therefore apple extracts can be used as an additive for removing bad breath or used in chewing gum and other products to remove the odor [43].

Apple polyphenols have a radiation protection effect by inhibiting radiation-mediated degradation of 2-deoxyribose (2-DR) in a dose-dependent manner. Apple polyphenols also reduce the damage caused by radiation through scavenging hydroxyl free radicals, which can inhibit single- and double-strand breaks induced by radiation in mouse thymocytes [44]. Studies on the weight-loss effect of apples have shown that apples have the potential to induce weight-loss. The apple polyphenols could reduce visceral fat, and the procyanidin oligomers in apple polyphenols can inhibit pancreatic lipase activity in the body, which lead to suppression of triglycerides. Apple polyphenols also help to inhibit glucose transport, decrease blood glucose levels and increase satiety. These findings indicate that apple indeed has a vital effect on losing weight [45]. It displays a certain medical and economic significance to design weight loss health products by apple polyphenols. Apple contains polyphenols, including anthocyanins, hydroxycinnamic acids, dihydrochalcones, flavonols, and flavan-3-ols. However, procyanidins were found at their maximum in apple (40–89%) [17]. Dihydrochalcones containing Phloretin (Phlor) and phloridzin (Phldz) possess anti-cancer, anti-obesity, anti-diabetic, antioxidant, anti-ageing, stress, hyperglycemia, anti-microbial, and melanogenic activity and have the efficiency to counteract liver, lung, and intestinal inflammation [46]. Due to the antifungal property of phloridzin and its derivative F2 against pathogen *Microsporum canis,* they are found to be more suitable in dermo-cosmetic applications [47]. They have many applications in food additives, beverages, membrane permeability and longevity extending agents in the food, cosmetic, and pharmaceutical industries related to phloridzin and its derivatives [48]. Ursolic acid (UA) (3β-hydroxy-urs-12-en-28-oic-acid), is a pentacyclic triterpenoid carboxylic compound (C30H48O3) that possess a wide range of pharmacological activities. It shows anti-cancer, hypoglycemic, analgesic, gastroprotective, anti-inflammatory, anti-hyperlipidemic, hepatoprotectory, anti-ulcer, anti-HIV, anti-atherosclerotic anti-androgenic, cardiovascular, antibacterial, antioxidant, diuretic, and cyanogenic activity [49]. It is found in plants, especially in the peel of fruits and leaves’ coating, such as apple fruit, eucalyptus leaves, rosemary leaves, and vinca leaves [50].

## 3. Polysaccharides (pectin)

**Figure 2:**
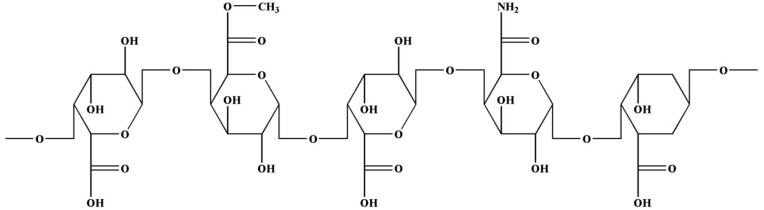
Structure of apple polysaccharide.

Apples contain a large number of polysaccharides (Figure 2) including pectin. The main components are glucuronic acid, lactose, arabinose, and glucuronic acid. Its basic structure is polygalacturonic acid [41,51]. The molecule contains neutral polysaccharide side chains, mainly L-arabinose, D-galactose, and L-rhamnose [52]. It is a natural polymer compound with good gelling and emulsifying stability. Apple pectin also has certain biological activities and has been widely used in medicine. 

Meanwhile, as a dietary fiber, apple pectin is an essential nutrient in people’s daily lives. Eating apples often have the effect of promoting digestion [39]. The dietary fibers in apples are divided into soluble and insoluble. Studies have shown that the proportion of dietary fiber contained in different types of apple averages about 1.79%, of which soluble dietary fiber accounts for about 0.4% and insoluble dietary fiber accounts for about 1.39%. The dietary fiber content of apple peel is 2 to 3 times higher than that of apple pulp [53]. 

Ohkami et al. (1995) found that apple pectin can significantly inhibit the proliferation of cancer cells in rats. If 20% of pectin is added into the diet and feed rate, the incidence of colon cancer was significantly lower than the control group, suggesting that apple pectin can effectively prevent and suppress colon cancer [54]. The dietary fiber and other nutrients contained in apples have been proved to elicit anti-cancer effects on the human body. In particular, it has a significant inhibitory effect on Hep G2 human liver cancer cell, MCF-7 human breast cancer cell, and CaCo-2 human colon cancer cell [55]. Janet et al. (1997) have shown that apple pectin reduces blood medium cholesterol, triglycerides, and high-density lipoproteins. It has significant effects in promoting fat metabolism [56]. Nesterenko and co-workers in 2004 conducted a nuclear radiation experiment and found that the addition of apple pectin to the daily diet can significantly exclude radioactive substances in the human body [57]. Amit and Ghanshyam (2010) found that apple pectin can inhibit the absorption of fat, suggesting that apple pectin can be used as a health food for weight loss and has a good application prospect [9]. Tomohiko et al. (2009) found that quercetin has anti-tumor, antibacterial, anti-inflammatory, antiviral, and hemostatic effects. Animal experiments have found that long-term intake of apple pectin can enhance the intestinal absorption of quercetin [58].

## 4. Phytosterols

**Figure 3:**
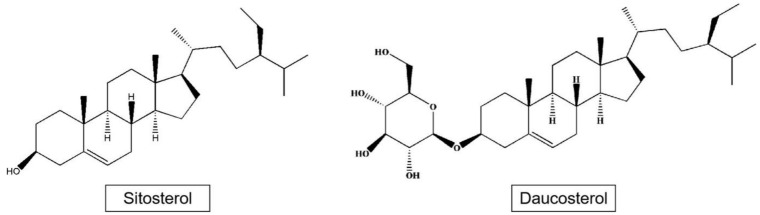
Structures of sitosterol and daucosterol.

Phytosterols such as sitosterol and daucosterol have been isolated from apples (Figure 3). Phytosterols are widely found in the roots, stems, leaves, fruits, and seeds of plants, and are part of the plant cell membrane. The content of plant sterols in apple seeds reaches 3.8 mg/g, mainly including β-sitosterol, stigmasterol, and campesterol, which is a kind of high-quality oil raw material with great development potential [34].

Phytosterols have strong anti-inflammatory effects including treatment of periodontitis, oral cavity ulcers, and other related diseases. β-sitosterol has a strong anti-asthma, cough, expectorant effect and can promote the repair of chronic bronchitis. Phytosterols can inhibit the synthesis and absorption of cholesterol, and promote the metabolism of cholesterol. For example, the daily intake of several hundred milligrams of phytosterols has a certain effect on reducing cholesterol in Japan [59]. Phytosterols can also prevent coronary atherosclerosis and treat heart disease. Prevention and treatment of skin squamous cell carcinoma and cervical cancer by phytosterols have beneficial effects [60]. Also, phytosterols can accelerate the rate of wound healing, enhance capillary circulation, promote muscle proliferation, and prevent the formation of gallstones [61]. Phytosterols can also be used to produce vitamin D3 and steroid drugs. Because of high permeability on the skin, phytosterols can maintain skin surface moisture, keep the skin tender, and reduce skin diseases. Similar to polyphenols, phytosterols also have good antioxidant properties and can be used as natural food antioxidants [62].

## 5. Pentacyclic Triterpenes

**Figure 4:**
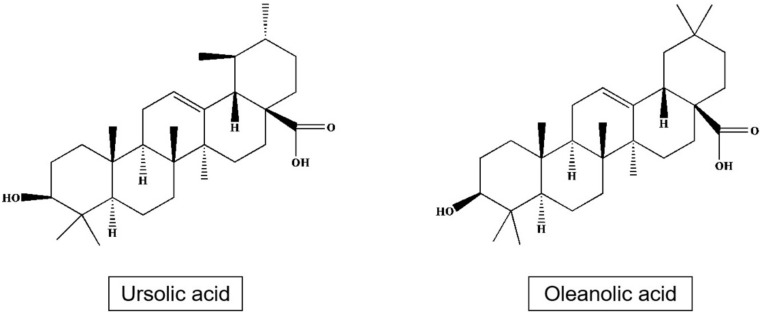
Structures of ursolic acid and oleanolic acid.

Triterpenes and especially pentacyclic triterpenes form a significant part of bioactive substances of apple [63,64]. These mainly include triterpenic acid, ursolic acid, 2α-hydroxy Ursan, 3β, 20β-hydroxy Ursan-28-oic acid, oleanolic acid, 2α-hydroxy oleanolic, betulinic, 3-O-p-coumaroyl 3-O-p-kumaroyl torment, and other [65], or 3β-trans-cinnamoyl oxy-2α-hydroxy-urs-12-en-28-oic, which is one of the main components of the apple peel [66] (Table 5; Figure 4).

Similarly, like apple polyphenols, pentacyclic triterpenes also exhibit many biological effects out of which the most significant is their cytotoxicity [67], which makes them potential drugs of tumor diseases [68]. Ursolic acid and particularly 2α-hydroxy-ursolic acid isolated from the apple peel inhibits the growth of tumor (HL-60, BGC, Bel-7402 and Hela) cell lines [69]. Pentacyclic triterpenes are currently one of the promising groups of secondary plant metabolites that exhibit remarkable effects in the prevention and therapy of malignancies [70]. Nowadays, the treatment of cancer is not only a question of the elimination of tumor cells by the induction of apoptosis. New therapeutic strategies also modify the microenvironment of the tumor to prevent angiogenesis, modulation of the immune response, or chronic inflammation, which is often associated with cancer. Especially triterpenes of lupine, oleanolic, and ursolic type [71], which are all richly represented in the apple peels, are very promising in this respect [34].

Most of these triterpenes exhibit a high anti-tumor potential with significant differences in human tumor cell lines. Acid 2α-hydroxy-ursolic,2α-hydroxy-3β-{[(2E)-3-phenyl-1-oxo-2-propenyl] oxy} Olean-12-en-28-oic acid and the 3β-p-trans-2α cumaroyloxy-hydroxyolean-12-en-28-oic, exhibited a higher anti-proliferative activity against the tumor cells of HepG2. Ursolic acid, 2α-hydroxy-ursolic, and 3β-trans-beta-cumaroyloxy-2α-hydroxyolean-12-en-28-oic exhibited higher anti-proliferative activity against the cancer cells of MCF-7. The anti-proliferative activity against the cancer cells of Caco-2 was exhibited by all pentacyclic triterpenes isolated from the apple peel, particularly by 2α-hydroxy-ursolic acid, maslinovo acid, 2α-hydroxy-3β-{[(2E)-3-phenyl-1-oxo-2-propenyl] oxy} Olean-12-en-28-oic acid, and the 3β-trans-beta-cumaroyloxy-2α-hydroxyolean-12-ene-28-oic, which had the highest level of the anti-proliferative activity [34].

Pentacyclic triterpenes, however, exhibit other interesting effects on the humans. For example, ursolic acid has significant anabolic effects on skeletal muscles, which plays a vital role in the process of ageing [72]. The same study has shown that ursolic acid strengthens neomyogenesis by increasing the number of satellite cells, and it increases the performance of skeletal muscles. Therefore it is a suitable candidate for the treatment of pathological states connected with muscle atrophy and muscle dysfunction, including atrophy of skeletal muscles, amyotrophic lateral sclerosis (ALS), sarcopenia, and metabolic diseases of muscles [72].

## 6. Other Nutrients

Richardson et al. (2020) reported in their study that apple contains multiple vitamins including vitamin C, E, β-carotene and essential mineral elements such as calcium, iron, potassium, manganese, zinc, magnesium, copper, and sulfur [73]. Vitamin C is also a strong antioxidant, and the amount present in 100 g of apple is 5.7 mg [2]. The mineral content mg per 100 g apple are K (107.25), Ca (5.80), P (10.87), Mg (5.07), Na (0.72), Fe (0.123), Zn (0.043), Cu (0.027), and Mn (0.035) [74].

Besides, the apple plants also contain some essential plant endogenous hormones, including auxin, gibberellin, cytokinin, abscisic acid, and ethylene [75]. The vitamin C contained in apples can enhance human immunity, prevent pernicious anaemia, and also has good antioxidant and anti-ageing effects. Apple contains zinc, which is an indispensable element that constitutes nucleic acids and proteins that are closely related to memory. Zinc deficiency causes children’s hippocampus to develop poorly [43]. Apple seeds also have high nutritional value, which is rich in protein and fat. The average fat content of different varieties of apple seeds is about 21.98%, which is 4.92 times that of chestnut. The average protein content is 38.53%, which is 4.64 times that of chestnut. The fatty acid composition of apple seeds shows that the potentially dominant compounds are oleic acid (46.5%) and linoleic acid (43.81%). The physical and chemical properties of apple seed oil are comparable to edible oils, indicating that it has better stability and potentially used in the food and pharmaceutical industries. Apple seed oil has the characteristics of high iodine and saponification value. Apple seed oil may be a good source of natural antioxidants and its antioxidant capacity (IC_50_) 40.06 μg/mL. Moreover, the in vitro cytotoxic activity against specific cancer cell lines shows its potential as an anticancer agent [76]. The cytotoxicity of apple seed oil to CHOK1 (Chinese hamster), SiHa (human cervical cancer cell), and A549 (human lung carcinoma) cancer cell lines are between 0.5% ± 0.06% and 88.6% ± 0.3% [77].

## 7. Apple-derived Products and Prospects 

As mentioned above, apple polyphenols have multiple medical and healthcare functions. Therefore, it can be used in the development of healthy foods and beverages with unique functions. For example, apple juice can be developed as a functional food that assists in suppressing tumors, lowering blood pressure, anti-ageing, losing weight, or nutritional products for improving growth, memory, and sleep quality. At present, there are already malic-acid-containing nutrition tablets and apple soft capsules on the market, which can be used to weight control and moisturize the skin. El-Messery and co-workers extracted polyphenol compounds from apple peels and added them to yogurt after microencapsulation. It not only guarantees the various health benefits of apple polyphenols, but also avoids the bioavailability and total acceptability of yogurt after it reacts with the ingredients of yogurt itself [78].

Pectin has always been a natural ingredient in human food. Pectin can be extracted from apples and used as a gelling agent, thickening agent, tissue forming agent, emulsifying agent, and stabilizer in food. Besides, the pectin in apples has a strong adsorption function for lead, mercury, and other harmful materials, suggesting that it has a good detoxification effect [79]. Apple pectin is commonly used in the manufacture of jams, jellies, preserved fruits, and juice drinks in food. In medicine, apple pectin can also be used as an adjunct to pharmaceutical preparations. In other industries, apple pectin is prepared as a film that is biodegradable and easy to recycle [80]. Also, bright pigments can be extracted from apple peels, which can be used to color acidic foods. It is a natural food pigment with development potential.

Triterpenoids in apples have various activities such as anti-tumor, anti-oxidation, and immunity enhancement, but it is difficult for traditional edible methods to make full use of triterpenes [34]. At present, the development and utilization of triterpenoids are insufficient, and the beverages with triterpenes have not yet been seen. As apple triterpenes have a particular bitter taste, adding other plant-derived sweeteners can adjust the sugar-acid ratio of the beverage and improve the taste of the beverage [9]. Compounding konjac gum and xanthan gum can increase the stability of the beverage. Functional beverages have huge market demand, and the development of an apple triterpene beverage has broad prospects [81]. With the rapid development of the fruit juice processing industry, the discharge of pomace is gradually increasing. Apple pomace is acidic and piled together will quickly ferment and deteriorate, which wastes resources and pollutes the environment. Using biological fermentation technology and suitable combination of microbial composite strains, the nutritional value of the peel and pomace after biological fermentation is greatly improved and has advantages in food products [82]. In addition to the significantly increased crude protein content, the product also contains beneficial micro-ecological factors, which has become an excellent green biological feed with dual characteristics of protein feed and micro-ecological preparation. In some animal studies, Skinner et al. (2018) have shown that apple pomace has the potential to be a safe livestock feed additive and its concentrations within the established safety thresholds for human consumption. When compared to many other commonly consumed fruits and veggies in countries like the United States, apples had the second-highest level of antioxidant activity. Apples are considered second after cranberry for the total concentration of phenolic compounds. However, apples could be considered a classical healthy diet as they contain the highest portion of free phenolics when compared to other fruits such as strawberry, cranberry, red grape, pear, peach, orange, pineapple, banana, lemon, and grapefruit [2]. Commercial development of apple pomace for human consumption requires more research focusing on standardized methods of nutrient reporting, mechanistic studies, and human clinical trials in the future [83]. Yet, the presence of phytochemicals (flavonoids) in a vast number of apples or its products can be a significant source of these nutrients in average daily diets of a person in Europe [2].

## 8. Traditional Uses 

Since ancient times, except seeds, whole fruit is edible and is used for making jams and cakes. However, apples have at least partially instigated the adage ‘An apple a day keeps the doctor away.’ Apples can cure different ailments, such as asthma, acidity, arthritis, diarrhoea, fever, obesity, headache, stomach aches, skin diseases, and respiratory problems [84]. The use of apple vinegar helps in treating anaemia as it contains iron in a very well-digestible form, as vitamin B12 and folic acid. Also, apple cider vinegar is effective in asthma, stone-kidney, arthritis, and skin diseases [85].

## 9. Medicinal Uses 

The chemical compounds present in apple are of great medicinal uses. The apple, as well as vinegar obtained from apple juice, has several medicinal values [86]. Apple cider vinegar has a significant property to cure many diseases related to human beings, and it normalizes the activity of the nervous system while also increasing blood clotting, which controls the effect of blood loss and improves the work of blood vessels. It is also considered as a source of nutrient and vital energy. It strengthens the gums and muscle of the heart, and vascular walls contribute to the normalization of the gastrointestinal tract [85]. Cysteine, malic acid, and arginine present in apple are considered appropriate to remove the stored toxic substance out from the body. These contents are also found to be effective against gout, uric acid, urticarial, and for the treatment of kidney-related diseases [87]. Stable water in oil emulsion containing extract of apple juice 3% formulation applied on hyper-pigmented human skin is considered useful to reduce sebum production, decreased melanin level, greasiness, and erythema causing acne, improving the appearance of oily facial skin [88].

### 9.1. Pharmacological Studies

Different types of the bioactive compound have been identified from apple fruit (*Malus* spp.). These bioactive compounds possess remarkable medicinal value as well as on the animal model; the peel and fruit juice extract study shows many pharmacological activities, which are considered as beneficial for human health. 

#### 9.1.1. Antioxidant Activities 

The flavonoids and phenolics compounds present in apple shows biological activities as an antioxidant, antimicrobial and enzyme inhibitory effects (against cholinesterase, tyrosinase, amylase, and glucosidase). Studies reported that the apple peels show good activity than pulps [89]. It has been reported that water, alcohol, and polyphenol extracts of *M. domestica* fruit were found to be most effective against gram +ve and gram –ve bacteria such as *B. subtilis, S. aureus, S. epidermidis, K. pneumonia*, *E. coli,* and *P. aeuroginosa,* respectively, which could be usefully applied to the food, pharmaceuticals, and cosmetics industries [90,91]. It has been found that quercetin compound is abundantly present in apple peel, acts as an antioxidant, and the apple’s potential antioxidant activity was approximately 83µmol vitamin-C equivalents, which means that the antioxidant activity of 100 g apples is equivalent to about 1500 mg of vitamin-C [4,92].

#### 9.1.2. Anti-inflammatory Activity 

It has been found that various triterpene acids such as chlorogenic acid and maslinic acid have potential in anti-inflammatory activity. Maslinic and pomolic acids show anti-inflammatory and anti-arthritic effects through NF- ĸB inactivation. Some researchers found that many significant sources of such compounds show potential in anti-inflammatories and glycemic control effects. Graziani and co-workers also reported that polyphenol extracts of apple prevent damage to human gastric epithelial cells in vitro and rat gastric mucosa in vivo [93].

#### 9.1.3. Cholesterol-Lowering Effect 

In rat males, *M. domestica* supplementation effect showed that there was a reduction in the amount of total cholesterol, LDL, and triglycerides and increased HDL concentration due to the presence of antioxidant compounds constituting in their diet results to inhibition of lipid peroxidation [94]. Foods that include dietary fiber abundantly and low in energy density promote healthy weight maintenance or weight loss. Apple contains a high amount of dietary fiber. In Brazil, a study was conducted on 49 overweight women with high blood cholesterol levels to determine the effect of fruit intake on blood lipids and body weight (70). The women were assigned to one of three diet groups for 12 weeks; the first group included a daily intake of 300 g of apple (~1.5 large size), the second group has a daily intake of a similar quantity of pear, and the third group had an intake of 60 g of oat cookies. All three groups were given additional dietary fiber provided by each of the treatments.

Guidelines were provided in each group for a moderately hypocaloric diet designed to reduce body weight at a rate of 1 kg/mo (deficit of 250 kcal/d). Study results revealed two reports. The addition of apples as part of average daily caloric intake of 2401 ± 389 kcal resulted in a significant weight loss of 1.32 kg after 12 weeks. Moreover, it has been concluded that the weight loss was due to the significant decrease in the energy density of the die; in addition, apples led to a decrease in blood glucose level compared to the oat cookies [95]. Vidal and coworkers (2005) suggested that apple polyphenols extract decrease plasma lipid levels by inhibiting Apolipoprotein B (apoB) secretion by inhibiting apoB synthesis without increasing the degradation of the newly synthesized protein and reduce the esterification of cholesterol in Caco-2/TC7 cells [96]. The presence of major compounds such as catechin, epicatechin, and procyanidin B1 polyphenols in apple varieties showed a cardiovascular protective effect on rats and resulted in lowering cholesterol in the blood [97].

#### 9.1.4. Antidiabetic Activity

Diabetes mellitus, commonly known as diabetes, is a metabolic disorder that is principally characterized by insulin resistance, relative insulin deficiency, and high blood sugar level. Very few information is available regarding apple and its polyphenol extracts potential effect on regulating glucose levels in the blood and other markers related to diabetes. It has been found that in apple extract, two inhibitors at concentrations 10 times dilution (I_2_) showed minimum absorbance resulting in the reduction in browning at a glucose concentration. So, in diabetes, *M. domestica* is effective and efficient in reducing the level of glycation in conditions [98]. Due to the presence of a higher amount of quercetin, apple consumption may lower the risk for diabetes, and its peels were also associated with a decreased risk in type II diabetes. Phenolic compounds including flavonoids present in apple juices were found to affect insulin, plasma concentrations of glucose significantly, and another two hormones, glucose-dependent insulinotropic polypeptide and glucagon-like peptide-1, in volunteers, and the result appeared to be consistent with delayed intestinal absorption of glucose [2].

Flavonoids played a significant role in the pharmacological actions in vivo and in vitro [99]. Also, these are associated with health-promoting effects and are beneficial in medicinal, nutraceutical, and cosmetic applications [100]. Flavonoids obtained from apple peel extracts have been reported effective in hypertension and cardiovascular disease [101]. The major types of flavonoids present in apple and its products are flavonols, flavanols, or catechins and anthocyanins with the leading representatives (-)- epicatechin, quercetin glycosides, and cyanidin galactoside, respectively [88].

#### 9.1.5. Anticancer Activity

It has been suggested that consuming one or more apple daily helps in reducing the risk of cancer compared to consumption of less than one apple per day [102]. In animal model studies, apples have been found to be effective in preventing skin, mammary, and colon carcinogenesis, while epidemiological observations indicated that regular consumption of one or more apples a day might reduce the risk for lung and colon cancer [2,103]. Jedrychowski and coworkers studied four different fruits including berries, stone fruits, citrus, and apple, in which on apple was found more specific and significant in reducing (63%) of colorectal cancer risk [104]. 

Apple extracts display dose-dependent anti-cell proliferation activity in Caco-2 colon cancer and Hep G2 liver cancer cells and shows maximum inhibition of 43% and 57% at 50 mg/mL, respectively. It was also concluded that due to a higher amount of phytochemical nutrients in apple peel, peels alone have more efficiency in inhibiting Hep G2 cell proliferation than whole apple [4]. In studies, it has been found that polyphenols present in apples played a significant role in affecting signalling pathways that control growth, cell survival, and proliferation both in vitro and in vivo. Phloretin has the efficiency to inhibit human hepatoma (Hep G2) and human colorectal cancer cells through inhibition of type 2 glucose transporter (GLUT2). This inhibition of intracellular glucose uptake was the main reason behind killing the cancer cell because these cancer cells depend on aerobic glycolysis for energy production. Also, in colorectal cancers, nuclear factor-kB (NF-kB) activate through lipopolysaccharide (LPS) that binds to the Toll-like receptor 4 (TLR4). Modification of polysaccharide components present in apple changed the LPS/TLR4/NF-kB pathway; consequently, supplementation of apple polysaccharide significantly inhibited the migratory ability in vitro on the LPS/ TLR4/NF-kB pathway in colorectal cancer cells (HT-29 and SW620 cells). Also, it has been reported that consumption of one apple in a day reduces the risk of colorectal cancer and consumption of more than one apple daily reduce the risk of colorectal cancer by 50% [102].

## 10. Conclusions

Apple and its peel are associated with good human health and experimental evidence from in vitro and in vivo studies indicative of its positive role in the prevention and treatment of diseases. Our review supports all the previous studies that have been reported. However, in many reported review articles, the literature is very insufficient. Our review focused on animal model studies, which are suitable as per the exact amount of concentration reported. In both in vivo and in vitro studies, the efficacy of nutrients present in apple against diseases was evaluated. At present, there is still a great prospect for the development and utilization of bioactive substances in apple. The consumption of apple and its processed products or extracts rich in polyphenols has been linked to reduced risk in cancer, cardiovascular disease, diabetes, and many other chronic diseases, including asthma. Polyphenols exert these health effects through antioxidant and anti-inflammatory activities and by modulating biomarkers in various cell signalling pathways. While sufficient evidence has been found for some of the health beneficial effects, many of them still require further studies. Further research should focus on the extraction of more apple bioactive substances and the development of drugs and functional food that can prevent tumor, inflammation, and cardiovascular disease. It will not only contribute to the protection of human health, but also provide economic benefits.

## Figures and Tables

**Table 1:** The distribution of bioactive substances in apple.

Bioactive Substance	Presence	References
Cinnamic acid species	peel	[3]
Chlorogenic acid	peel, pulp, kernel, leaf	[4]
Caffeic acid	peel, pulp	[4]
Ferulic acid	pulp, leaf	[5]
P-coumaric acid	pulp	[5]
Caffeoylquinic acid	pulp and skin	[3]
P-coumarylquinic acid	pulp and skin	[3]
Cinnamic acid	leaf	[5]
(+)-Catechin	peel, pulp, kernel	[6]
(-)-Epicatechin	peel, pulp, kernel	[6]
Proantho Cyanidins	peel, pulp, leaf	[6]
Flavonols		[7]
Quercetin and quercetin glycosides	peel	[4]
Anthocyanins	peel	[7]
Phytosterol	peel	[8]
Triterpenoids	peel	[8]
Pectin	peel, pulp, root, stem, leaf	[9]
Carbohydrate	seed, pulp, leaf	[10]
Amino acid, peptide and protein ingredients	seed, pulp	[10]
Oil composition	seed, pulp	[10]
Endogenous hormone	Plant	[10]

**Table 2:** The lists of phenolic acids isolated from apples.

Structure	References
Salicylic acid	[4]
P-coumaric acyl quinic acid	[4,5]
D-(-)-quinic acid	[5,6]
Chlorogenic acid	[4,5]
Caffeic acid	[6]
Ferulic acid	[6]
2(R)-hydroxybutanedioic acid	[4,6]
2(R)-hydroxybutanedioic acid 1-methyl ester	[4]
Malonic acid	[6]
Maleic acid	[6]
D-(-)-quinic acid	[5,6]
Chlorogenic acid	[4,5]
Caffeic acid	[6]
Ferulic acid	[6]
2(R)-hydroxybutanedioic acid	[4,6]
2(R)-hydroxybutanedioic acid 1-methyl ester	[4]
Malonic acid	[6]
Maleic acid	[6]
P-cournaric acid	[4,6]
Cinnamic acid	[4]

**Table 3:** The list of flavonoids isolated from apples.

Compound	References
Catechins	[7]
Epicatechin	[7]
Proanthocyanidins B1	[7]
Proanthocyanidins B2	[7]
Proanthocyanidins B5	[7]
Proanthocyanidins C1	[7]
Proanthocyanidin tetramer	[8]
Proanthocyanidin pentamer	[8]
Proanthocyanidin hexamer	[8]
Proanthocyanidin heptamer	[8]
Proanthocyanidin octamer	[8]
Phlorizin	[9]
Phloretin	[9]
Quercetin	[9]
3,5,7,3 ‘, 4’-pentaflavonol-3-o-xyloside	[10]
3,5,7,3 ‘, 4’-pentaflavonol-3-o-rhamnoside	[10]
3,5,7,3 ‘, 4’-pentaflavonol-3-o-galactoside	[10]
3,5,7,3 ‘, 4’-pentaflavonol-3-o-glucoside	[10]
3,5,7,3 ‘, 4’-pentaflavonol-3-o-arabinoside	[10]
3,5,7,3 ‘, 4’-pentaflavonol-3-o-rutinoside	[10]
Anthocyanidins	[10]
Ideain(cyanidin-3-galactoside)	[10]

**Table 4:** Concentrations (micrograms per gram of fresh weight) of phenolic compounds present in apple peel and flesh.

Compounds	Peel	Flesh	References
chlorogenic acid	136.1	177.3	[17]
*p*-coumaroylquinic acid	12.4	15.7	[17]
**total hydroxycinnamics**	148.5	193	[17]
cyanidin 3-galactoside	86.0	ND	[17]
**total anthocyanins**	86	ND	[17]
epicatechin	287.3	76.7	[17]
procyanidin B1	136.4	62.8	[17]
procyanidin B2	275.2	107.5	[17]
other procyanidins	185.3	ND	[17]
**total procyanidins**	958.2	267.7	[17]
phloretin 2′-xylglucoside	40.2	4.9	[17]
phloridzin	72.3	14.4	[17]
3-hydroxyphloretin 2′-xylglucoside	3.5	ND	[17]
3-hydroxyphloretin 2′-glucoside	7.7	ND	[17]
**total dihydrochalcones**	123.7	19.3	[17]
**total polyphenolics**	1604.4	481.3	[17]
**total phenolic content**	1323.6	429.6	[17]

Note: **ND** (not detected).

**Table 5:** The triterpenes isolated from the apple peel.

Compound	References
2α-hydroxy-3β-{[(2E)-3-phenyl-1-oxo-2-propenyl]oxy}urs-12-en-28-oicacid	[33]
3β-trans-cinnamoyloxy-2α-hydroxyurs-12-en-28-oic acid	[33]
3β-trans-p-coumaroyloxy-2α-hydroxyurs-12-en-28-oic acid	[33]
3β-cis-p-coumaroyloxy-2α-hydroxyurs-12-en-28-oic acid maslinic acid	[33]
2α-hydroxy-3β-{[(2E)-3-phenyl-1-oxo-2-propenyl]oxy}olean-12-en-28-oic acid	[33]
2α-hydroxy-3β-{[(2Z)-3-phenyl-1-oxo-2-propenyl]oxy}olean-12-en-28-oic acid	[33]
3β-trans-cinnamoyloxy-2α-hydroxyolean-12-en-28-oic acid	
3β-trans-p-coumaroyloxy-2α-hydroxyolean-12-en-28-oic acid	
3β-cis-p-coumaroyloxy-2α-hydroxyolean-12-en-28-oic acid	[33]
3β,13β-dihydroxyurs-11-en-28-oic acid	[8]
2α,3β,13β-trihydroxyurs-11-en-28-oic acid	[8]
3β,28-dihydroxy-12-ursene	[8]
olean-12-en-2α,3β-diol	
olean-12-en-3β-ol (β-amyrin)	
olean-12-en-2α,3β,28-triol	
urs-12-ene-2α,3β-diol,	[8]
urs-12-ene-3β-ol	[8]
urs-12-ene-2α,3β,28-triol	[8]
3β-trans-p-coumaroyloxy-2α,3β,13β-trihydroxy-urs-11-en-28-oic acid	[8]
3β-cis-p-coumaroyloxy-2α,3β,13β-trihydroxy-urs-11-en-28-oic acid	[8]
urs-12-en-28-ol	[8]
